# When Specialists Are Scarce: What a Decade of Trials Teaches Us About Child Development in South Asia

**DOI:** 10.1192/bjo.2026.11147

**Published:** 2026-06-30

**Authors:** Tooba Akhtar, Ahmed Waqas, Kashif Zaman, Ainy Gul, Atif Rahman

**Affiliations:** 1 Trinity College Dublin, Dublin, Ireland; 2 University of Liverpool, Liverpool, United Kingdom; 3 Greater Manchester Mental Health NHS Foundation Trust, Prestwich, United Kingdom; 4 Wuhan University of Science and Technology, Wuhan, China; 5 North West School of Psychiatry, Manchester, United Kingdom

## Abstract

**Aims:**

South Asia bears a substantial burden of developmental and psychosocial adversity among children and adolescents, yet specialist mental health resources remain scarce. Scalable, non-specialist delivered interventions have been proposed as a solution, but the breadth, characteristics, and evidentiary foundations of such interventions across developmental stages remain poorly synthesised.

This review aimed to systematically examine the existing evidence on child-centred interventions delivered by non-specialists in South Asia, including whether such interventions demonstrate successful outcomes, are predominantly preventive or promotive in focus, are developmentally targeted, and are implemented with acceptable participant retention across diverse settings.

**Methods:**

A scoping review was conducted in accordance with Preferred Reporting Items for Systematic Reviews and Meta-Analyses guidance (PRISMA). Web of Science and PubMed were searched for peer-reviewed intervention studies published from 2010 onwards. Eligible studies were clinical trials with a comparison group, conducted in South Asia, targeting infants, children, or adolescents up to 19 years, and aiming to improve developmental or wellbeing outcomes through prevention, promotion, or treatment approaches. Pharmacological studies, qualitative designs, and samples with high baseline psychiatric morbidity were excluded. Screening, full-text review, and data extraction were undertaken independently by multiple reviewers with consensus resolution.

**Results:**

Forty-seven studies met inclusion criteria, encompassing 57,951 participants at baseline and 49,321 at endline, with an overall attrition rate of approximately 15%. Nearly half of studies were conducted in India, followed by Bangladesh and Pakistan. Most interventions were randomised or cluster randomised trials and were delivered in schools, homes, or community settings. Non-specialists delivered 87% of interventions, including health workers, lay counsellors, peers, and teachers. Interventions predominantly focused on promotion (68%) or prevention, with only two treatment-focused trials.

Perinatal and early childhood interventions predominantly emphasised developmental stimulation (n=21), parenting practices (n=18), psychoeducation related to child health, development, nutrition, or maternal wellbeing (n=19), and responsive caregiving or maternal responsiveness (n=11). In contrast, adolescent interventions primarily targeted psychosocial wellbeing (n=8), positive youth development approaches (n=5), life skills, stress management, or resilience-focused outcomes (n=multiple studies; exact counts varied by domain).

Cognitive behavioural components were present in a minority of interventions (n=7 overall), most commonly embedded within multi-component programmes rather than delivered as stand-alone therapies. The use of standardised developmental and psychosocial outcome measures was common, with the Bayley Scales of Infant and Toddler Development (Second edition: n=6; Third edition: n=11) most frequently used for early childhood outcomes, and the Strengths and Difficulties Questionnaire (n=7) most commonly employed for child and adolescent psychosocial wellbeing.

**Conclusion:**

Over the past decade, South Asia has generated a substantial and methodologically robust body of evidence demonstrating the feasibility and acceptability ofnon-specialist delivered child and adolescent developmental interventions across settings and developmental stages. However, the literature remains heavily weighted toward promotion and prevention, with notable gaps in middle childhood and treatment-focused interventions. Future research should prioritise theoretically explicit, mechanism-informed designs and address under-represented age groups to inform scalable, equitable child mental health strategies in low-resource settings.

